# Probiotics and their role in gastrointestinal cancers prevention and treatment; an overview 

**Published:** 2018

**Authors:** Ahmad Javanmard, Sara Ashtari, Babak Sabet, Seyed Hossein Davoodi, Mohammad Rostami-Nejad, Mohammad Esmaeil Akbari, Azadeh Niaz, Amir Mohammad Mortazavian

**Affiliations:** 1 *Student Research Committee, Cancer Research Center, Shahid Beheshti University of Medical Sciences, Tehran, Iran.*; 2 *Basic and Molecular Epidemiology of Gastrointestinal Disorders Research Center, Research Institute for Gastroenterology and Liver Diseases, Shahid Beheshti University of Medical Sciences, Tehran, Iran.*; 3 *Department of Surgery, Faculty of Medicine, Shahid Beheshti University of Medical Sciences, Tehran, Iran*; 4 *Department of Clinical Nutrition, Faculty of Nutrition Sciences and Food Technology, Food Science and Technology, Shahid Beheshti University of Medical Sciences, Tehran, Iran*; 5 *Gastroenterology and Liver Diseases Research Center, Research Institute for Gastroenterology and Liver Diseases, Shahid Beheshti University of Medical Sciences, Tehran, Iran*; 6 *Cancer Research Center, Shahid Beheshti Medical University, Tehran, Iran*; 7 *University of Tehran, Tehran, Iran*; 8 *Department of Food Technology, Faculty of Nutrition Sciences and Food Technology, Shahid Beheshti University of Medical Sciences, Tehran, Iran *

**Keywords:** Probiotic, Prebiotics, Gastrointestinal cancer, Gut microbiota

## Abstract

Cancers of the gastrointestinal (GI) track are a serious global health problem. The human GI tract is home to trillions of microorganisms that known as gut microbiota and have established a symbiotic relationship with the host. The human intestinal microbiota plays an important role in the development of the gut immune system, metabolism, nutrition absorption, production of short-chain fatty acids and essential vitamins, resistance to pathogenic microorganisms, and modulates a normal immunological response. Microbiota imbalance has been involved in many disorders including inflammatory bowel disease, obesity, asthma, psychiatric illnesses, and cancers. Oral administration of probiotics seems to play a protective role against cancer development as a kind of functional foods. Moreover, clinical application of probiotics has shown that some probiotic strains can reduce the incidence of post-operative inflammation in cancer patients. In the present narrative review, we carried out update knowledge on probiotic effects and underlying mechanism to GI cancers. Currently, it is accept that most commercial probiotic products are generally safe and can used as a supplement for cancer prevention and treatment. Nevertheless, well-designed, randomized, double blind, placebo-controlled human studies are required to gain the acceptance of the potential probiotics as an alternative therapy for cancer control..

## Introduction

 Nowadays, besides of introducing the new technology and methodology for diagnostic and management of GI cancers, some additional aspects are becoming increasingly important, including the maintenance of health a counteracting cancers by health benefits of using probiotics and prebiotics in human nutrition. Probiotics are defined as living bacteria that, when consumed in sufficient quantities, carry the health benefits of the host (1). The main benefit of using probiotic is to help the host maintain the microbial balance of the intestine, reduce pathogenic gastrointestinal microorganisms, the improvement of bowel regularity, and the restoration of intestinal microbiota homeostasisin antibiotic-associated diarrhea (2). Furthermore, several studies have shown potential of probiotics in cancer prevention and treatment through microbiota modulation, immune modulation, reduced bacterial translocation, enhanced gut barrier function, anti-inflammatory and anti-pathogenic activity, with effects on reducing tumor formation and metastasis (3, 4). The possible association between probiotics and GI neoplasm has mainly been evaluated in relation to colorectal cancer (CRC) and gastric-cancer-related *Helicobacter pylori* (*H. pylori*) (5-9). These results indicate the probiotics are potential dietary supplements against neoplastic predisposition through extensive effects on the immune system of host (25–29). The current narrative review summarizes the update knowledge on probiotic effects and underlying mechanism to GI cancers. Moreover, we presented the comprehensive review of the evidence from clinical studies using probiotics in the prevention or/ and treatment of GI cancers. 

**Probiotics**

Most of the probiotic products currently available contain lactic acid bacteria (LAB) which belong to the* Lactobacillus* and *Bifidobacterium *(10). Some of the most important probiotic microorganisms that used in human nutrition are listed in Table 1. Most microorganisms recognized to date as probiotics are Gram-positive, with *Lactobacillus* and *Bifidobacterium *being the main species used as treatments of gastrointestinal disorders (11). However, some Gram-negatives are also used as probiotics. The best example of this group is *Escherichia coli *Nissle 1917 (EcN) (12), also known as “Mutaflor,” which has been used in recent years to treat chronic constipation and colitis in Germany (13, 14). Two of the most commercially important LAB that playing an important role in the dairy products are *Streptococcus thermophilus* and *Lactococcus lactis* (15).

**Selection criteria for probiotic strains**

According to the World Health Organization (WHO), Food and Agriculture Organization (FAO), and European Food Safety Authority (EFSA), probiotic strains must meet both safety and functionality for human and animal health, as well as to their technological usefulness (Table 2). Microorganism that used as probiotics should meet the terms of GRAS (Generally Regarded as Safe) and QPS (Qualified Presumption of Safety) status and the safety of a strain is defined as the absence of association with pathogenic cultures, and the antibiotic resistance profile. Functional aspects define their survival in the gastrointestinal tract and its safety effects (16, 17). Due to the rapid growth of the probiotic market, the probiotic survival and maintenance of their properties throughout the storage and distribution process is very important (18, 19). Finally, Suitable probiotic strains should have a positive effect on the host, transfer through the intestinal tract, adhere to the epithelial cells of the intestine, produce antimicrobial agents against the pathogen and stabilize the intestinal microflora (20).

**Probiotics and GI cancers**

Gastrointestinal cancer refers to malignant conditions of the gastrointestinal (GI) tract and other organs involved in digestive system which includes cancers of the esophagus, gallbladder, liver, pancreas, gastric, small intestine, colon and rectum (21). GI cancers are a major health problem, accounting for 25% of all cancers and 9% of all causes of cancer death in the world (22). Colorectal, gastric and esophageal cancers are the third, fourth and eighth most common cancers with 1.4, 1 and 0.45 million new cases in 2012, respectively (23). GI cancers are considered as multifactorial diseases, complex relationships between genetics, epigenetics, immunity, environmental factors, diet and lifestyle that can interact with the gut microbiota and functions during the tumor genesis and growth (24, 25).

**Table 1:** Probiotic microorganisms used in human nutrition

Type Lactobacillus	Type Bifidobacterium	Lactic Acid Bacteria	Other Microorganisms
*L. acidophilus* (a)	*B. adolescentis* (a)	*Enterococcus faecium* (a)	*Bacillus clausii* (a)
*L. amylovorus* (b)	*B. animalis* (a)	*Lactococcus lactis* (b)	*Escherichia coli* Nissle 1917(a)
*L. casei* (a), (b)	*B. bifidum* (a)	*Streptococcus thermophiles* (a)	*Saccharomyces cerevisiae *(boulardi) (a)
*L. gasseri* (a)	*B. breve* (b)		
*L. helveticus* (a)	*B. infantis* (a)		
*L. johnsonii* (b)	*B. longum* (a)		
*L. pentosus* (b)			
*L. plantarum* (b)			
*L. reuteri* (a)			
*L. rhamnosus* (a), (b)			

(a) Mostly as pharmaceutical products;

(b) mostly as food additives

**Table 2:** Selection criteria for probiotic strains

Criteria	Required properties
Safety	Human or animal origin Isolated from the gastrointestinal tract of healthy individuals History of safe use Precise diagnostic identification (phenotype and genotype traits) Absence of data regarding an association with infective disease Absence of the ability to cleave bile acid salts No adverse effects Absence of genes responsible for antibiotic resistance localized in non-stable elements
Functionality	Competitiveness with respect to the microbiota inhabiting the intestinal ecosystem Ability to survive and maintain the metabolic activity, and to grow in the target site Resistance to bile salts and enzymes Resistance to low pH in the stomach Competitiveness with respect to microbial species inhabiting the intestinal ecosystem Antagonistic activity towards pathogens Resistance to bacteriocins and acids produced by the endogenic intestinal microbiota Adherence and ability to colonize some particular sites within the host organism An appropriate survival rate in the gastrointestinal system
Technological usability	Easy production of high biomass amounts and high productivity of cultures Viability and stability of the desired properties of probiotic bacteria during the fixing process High storage survival rate in finished products Guarantee of desired sensory properties of finished products Genetic stability Resistance to bacteriophages

There has been an increased interest in the scientific community on the use of probiotic therapy for prevention and treatment of a number of GI disorders, including irritable bowel syndrome (IBS), inflammatory bowel diseases (IBD), pathogenic bacterial or viral infection, and antibiotic associated diarrhea (26, 27). There is also epidemiological evidence that supports a protective role of probiotics against cancer (28). Substantial research has demonstrated that probiotics possess anti-proliferative or pro-apoptotic activities in GI cancers, among which colonic cancer cells and gastric cancer cells were most commonly studied (29, 30). Several studies were performed on the health benefits of milk fermented with *Lactobacillus casei* and *L. acidophilus* and the results indicate the positive effects of these probiotics on increase of tumor cell apoptosis (31, 32). Previous studies indicated the anti-proliferative role of *L. rhamnosus* GG strain in both human gastric cancer cells and colonic cancer cells (33-35), while another probiotic product named *Bifidobacterium adolescentis* SPM0212 inhibited the proliferation of three human colon cancer cell lines including HT-29, SW 480, and Caco-2 (36). Other probiotic products or strains that exerted antitumor activities against human colon cancer cells included Bacillus polyfermenticus (37), *L. acidophilus* 606 (38), LGG/Bb12 (39), and LGG/*Bifidobacterium animalis* subsp.* lactis *(40). In addition, Cousin et al. reported that fermented milk containing *Propionibacterium freudenreichii* enhanced the cytotoxicity of camptothecin that was used as a chemotherapeutic agent for gastric cancer (41). 

**Table 3:** Clinical trials of probiotics interventions for prevention, post-operative complications and treatment of CRC

Type of intervention	Patients	Probiotic strain	Length of treatment	Outcome	Ref
Prevention	38 healthy patients	*L. rhamnosus*	4 weeks	Reduce of b-glycosidase activity by 10% and urease activity by 13%	(44)
	17 healthy patients	*RS *vs. *BF lactis*	4 weeks	Induced unique changes in fecal microflora but did not significantly alter any other fecal, serum, or epithelial variables.	(45)
	10 CRC and 20 healthy patients	*L. gasseri* (LG21)	12 weeks	A deterioration of the intestinal environment was observed in the colorectal cancer patients in comparison to the healthy controls, and the intestinal environment improved when probiotics was taken.	(46)
Prevention of post-operative complication	100 CRC patients undergoing surgery (placebo group/ probiotics group n=50/50)	*L. plantarum * *L. acidophilus* *B. longum*	16 days	Improvement in the integrity of gut mucosal barrier and decrease in infections complications	(8)
	124 CRC patients undergoing surgery (placebo group/ probiotics group n=80/84)	*L. acidophilus * *L. plantarum* *B. lactis BB* *S. boulardii*	15 days	Decreased the rate of all postoperative major complication, gene expression of TNF and circulating concentrations of IL-6 were under the control of SOCS3 in the probiotics group	(47)
	156 CRC patients undergoing surgery (placebo group/ probiotics group n=81/75)	*E. faecalis* *C. butyricum* * B. mesentericus *	15 days	Probiotic treatment reduce superficial incisional surgical site infections (SSIs) in patients undergoing CRC surgery	(48)
	60 CRC patients undergoing surgery (placebo group/ probiotics group n=30/30)	*B. longum* *L. acidophilus* *E. faecalis*	12 days	Faster recovery of bowel function, lower incidences of diarrhea, and slightly lower rate of bacteremia.in probiotic group	(49)
Chemotherapy and radiation therapy related toxicity	150 CRC patients undertreated	*L. rhamnosus* GG	24 weeks	Patients had less diarrhea, less abdominal pain, less hospital care, and had fewer chemo dose reductions due to bowel toxicity	(50)
	490 gynecological cancer and CRC patients	*VSL*#3 (a mixture of 8 probiotics)	From the 1st day of radiation therapy	Significant decrease of diarrhea (31.6 vs. 51.8%) and Severe diarrhea (1.4 vs. 55.4%)	(51)

**Colorectal cancer**

Colorectal cancer (CRC) is the third most common cancer worldwide with more than 1 million new cases annually and is responsible for death of more than 500,000 people (42). Evidence has shown that taking probiotics is a protective approach for proper maintaining of healthy gut microbiota and also reducing the risk of colon cancer risk (43). Contrary to many in-vitro and in-vivo studies in animal models and cancer cell lines of human, few randomized placebo-controlled trials (RCTs) studies have reported the effect of probiotics on prevention and inhibition of intestinal carcinogenesis (44-46). The benefits of probiotics are not only limited to the prevention of intestinal cancers, but they can also include the prevention of symptoms and complications in patients undergoing colorectal surgery for cancer and who receiving intestinal cancer treatment (8, 47-51). In table 3 we summarized the results of clinical trials studies regarding the effect of probiotics intervention for prevention or/and treatment of colorectal cancers. 

**Table 4:** Clinical trials using probiotics in association with combination therapy of *H. pylori* eradication

Patients	Probiotic strain	Length of treatment	Outcome	Ref
120 dyspeptic adults	*L. acidophilus* LB	10 days	Eradication rate ↑, side effects ↔	(93)
60 asymptomatic adults	*L. rhamnosus* GG	14 days	Eradication rate ↔, side effects ↓	(94)
120 asymptomatic adults	*L. rhamnosus* GG	14 days	Eradication rate ↔, adverse effects ↑	(95)
160 dyspeptic adults	*L. acidophilus* La5 *B. lactis* Bb12	4 weeks	Eradication rate ↑, adverse effects ↓	(96)
85 asymptomatic adults	*L. rhamnosus* GG *S. boulardii* *L. acidophilus* La5 *B. lactis* Bb12	2 weeks	Eradication rate ↔, adverse effects ↓	(97)
70 dyspeptic adults with resistant *H. pylori*	*L. casei* ssp *L. casei* DG	10 days	Eradication rate ↔, adverse effects ↓	(98)
86 dyspeptic children	*L. casei*	2 weeks	Eradication rate ↑, adverse effects ↔	(99)
40 dyspeptic children	*L. reuteri*	20 days	Eradication rate ↔, adverse effects ↓	(100)
138 dyspeptic adults with resistant *H. pylori*	*L. acidophilus* La5 *B. lactis* Bb12	4 weeks	Urease activity↓ during pretreatment, eradication rate ↑, side effects ↓	(101)
65 children	*B. animalis* * L. casei*	unclear	Eradication rate ↑	(102)
118 individuals	*L. rhamnosus LC* *P. freudenreichii* *B. breve*	4 weeks	Eradication ↔, urease activity ↓, gastritis and *H. pylori* colonization ↓	(103)
90 individuals	*L. reuteri*	6 weeks	Eradication rate ↑	(104)

↑ Increase,

↓ decrease,

↔ no effect

**Table 5:** Clinical trials of probiotics interventions in patients with severe acute pancreatitis (SAP)

Patients	Probiotic strain	Length of treatment	Outcome	Ref
45 SAP patients	*L. plantarum*	7 days	reducing pancreatic sepsis and the number of surgical interventions	(67)
25 SAP patients	*B. longum* *L. bulgaricus* *S. thermophilus*	7 days	The time of abdominal pain alleviation, serum amylase restoration, the incidence rate of complications and mean of hospitalization were significantly decreased in group treated with probiotics	(105)
62 SAP patients	*P. pentosaceus* *L. mesenteroides* *L. paracasei* *L. plantarum* with bioactive fibers	7 days	symbiotic may prevent organ dysfunctions in the late phase of severe acute pancreatitis	(66)
298 SAP patients	*L. acidophilus* *L. casei* *L. salivarius* *L. lactis* *B. bifidum* *B. lactis*	28 days	Probiotic prophylaxis with this combination of probiotic strains did not reduce the risk of infectious complications and was associated with an increased risk of mortality.	(68)
90 SAP patients	*P. pentosaceus* *L. mesenteroides* *L. paracasei* *L. plantarum* with bioactive fibers	unclear	Synbiotic supplements was associated with lower infection rate lower rate of surgical interventions, shorter ICU and hospital stay and reduced mortality rate	(106)
70 SAP patients	*B. longum* *L. bulgaricus* *E. faecalis*	14 days	Early enteral nutrition (EN) with addition of probiotics resulted in significant lowering of the level of pro-inflammatory cytokines, earlier restoration of gastrointestinal function, decrease of complications and shortening of hospitalization	(107)

**Figure 1:**
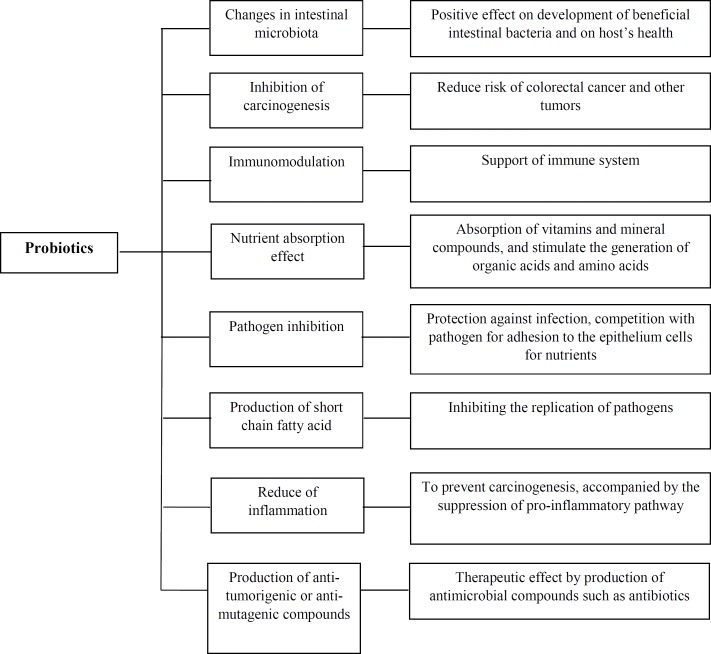
Anti-carcinogenic mechanisms of probiotics

The results of few clinical trial studies showed the effect of probiotics on manipulate the composition of gut microbiota, thus positively affect the host by improving intestinal barrier integrity, inhibiting growth of pathogens, reducing metabolism of pro-carcinogenic substances (44-46). Therefore, probiotics are effective in preventing and inhibiting the growth of intestinal cancer. In addition, several RCTs studies demonstrate that the use of probiotics in patients undergoing abdominal surgery is a promising approach to the prevention of post-operative superficial incisional surgical site infections (SSIs) and improvement in the integrity of gut mucosal barrier (47-49). Furthermore, the patients’ quality of life was also improved, shortening the duration of post-operative hospital stay and the period needed for antibiotics administration. Chemotherapy and radiotherapy as the conventional therapies for cancers can changes in the composition of the gut microbiota; these disruptions could also participate in the development of mucositis, particularly diarrhea and bacteraemia (52, 53). The prevention of cancer therapy-induced mucositis by probiotics has been investigated in randomized clinical trials with some promising results. Two trial studies on CRC patients who were undergoing chemotherapy and radiotherapy indicated a significantly decreased incidence of diarrhea by administration of *L. rhamnosus *GG and VSL#3 (a mixture of 8 probiotics) (50, 51). 

**Gastric cancer**

Gastric cancer (GC) represents the second cause of cancer-related death worldwide, accounting for approximately 10% of newly diagnosed cancers (23). Although GC incidence rate declined in recent last years, the 5 year survival rate of this neoplasm is under 25% with regional variations (54). Studies on probiotics and gastric cancer are mainly focused on eliminating Helicobacter pylori (*H. pylori*) infection as the major risk factors of gastric cancer (GC) (55). *H. pylori *is a Gram-negative bacterium which can disrupt the acid mucus barrier and colonize the gastric epithelium, is found in patients who are suffering from chronic gastritis, peptic ulcer and gastric cancer (56, 57). Inhibitory effects of probiotics on *H. pylori* infection have been observed in several animal models containing *B. bifidum, L. acidophilus, L. rhamnosus, L. salivarius* and several other probiotic strains (58). 

In recent years, the success of eradication therapies of *H. pylori *by combination therapy of proton pump inhibitor (PPI) and two antibiotics therapy (clarithromycin plus amoxicillin or metronidazole) has been declined, due to the development of *H. pylori* resistant strains. According to recently meta-analysis, using probiotics as a supplementation with antibiotic therapy is very useful to the *H. pylori *eradication (59-61). In table 4 we summarized the results of clinical trials studies regarding the effect of probiotics in association with antibiotics treatment in eradication of *H. pylori* colonization. The results of these studies suggest that probiotic supplementation during antibiotic therapy to *H. Pylori* eradication, decreases adverse side effects, resulting in better compliance and, in some cases, improved rates of eradication. In addition, gastric tumor promoting proliferation of lymphoid tissue disappeared after successful eradication (62, 63). One of the proposed mechanisms for probiotic treatment is that these microbes can be present in the stomach and even live in it temporarily, increase the immune response and reduce the effect of H. pylori inflammation on the host gastric mucosa (64). 

**Other GI cancers**

Unlike many studies on CRC and GC, there are few studies that suggest probiotic role in the prevention and treatment of other GI cancers such as pancreas and liver cancer. Pancreatic cancer is the 12th most common cancer in the world with 338,000 new cases and 7th most frequent cause of death worldwide with 331,000 deaths per year, but the etiology is still unknown (23, 65). Some previous studies supports a multifaceted role of probiotics in preventing pancreatic cancer by modulating pancreatitis and other risk factors such as diabetes, pancreatic necrosis, inflammation and obesity (66-68). Table 5 shows the results of clinical trials studies regarding the effect of probiotics on severe acute pancreatitis (SAP). The results of meta-analysis on six clinical trial studies indicate that probiotics did not significantly effects on the clinical outcomes of patients with SAP (69). Therefore, the available data are not sufficient to draw conclusions about the effects of probiotics in pancreatic cancer because of the limited number of trials and their heterogeneity. The types of probiotics and treatment strategies are very important in the heterogeneity of clinical outcomes reported in different RCTs.

Liver cancer is the fifth most common cancer in men and the ninth in women and is the second most common cause of death from cancer worldwide, estimated to be responsible for nearly 746,000 deaths in 2012 (23). The gut microbiome has been related to the development of liver disorders such as liver fibrosis (70), non-alcoholic fatty liver disease (71) and more recently, liver cancer (72). In the previous year, it was reported that probiotics inhibit hepatocellular carcinoma (HCC) progression in mice (73). Feeding a probiotics mixture to tumor-injected mice could shift the composition of gut microbiota and reduce the size of liver tumors. In addition to the reduction of tumor size, angiogenic factors were down regulated by probiotics administration.

**Anti-carcinogenic mechanisms of probiotics on GI cancers**

Theoretically, probiotics are able to reduce cancer risk by several mechanisms. Oral administration of probiotics has multiple effects such as normalization of gut microbiota, improvement of the gastrointestinal barrier, inhibition of potential pathogens, anti-inflammatory activities and suppression of tumor formation and growth. Figure 1 presents a scheme of the possible anti-carcinogenic mechanisms of probiotics.

Probiotics have abundant anticancer benefits and have a major impact on the quantitative and/or qualitative changes of the intestinal microbiota. The intestinal microbiota has been linked to GI cancer development also by production of toxic and genotoxic bacterial metabolites that can lead to mutations by binding specific cell surface receptors and affecting intracellular signal transduction. Specific strains of bacteria are involved in the pathogenesis of cancer, including *Streptococcus bovis*, *Bacteroides*, *clostridia*, and *H. pylori *(74-76). On the contrary, some bacterial strains, including L. acidophilus and B. longum, inhibit carcinogenic tumor growth in the colon (77, 78). Thus, a balance between “detrimental” and “beneficial” bacteria has implications in setting the stage for cancer. Shifting the proportion of microbes has been reported to influence carcinogen bioactivation and thus cancer risk. It is increasingly apparent that dietary components can significantly modify this balance. In addition, probiotics also affect the intestinal microbiological compositions, thus positively affect the host by improving intestinal barrier integrity, inhibiting growth of pathogens, reducing metabolism of pro-carcinogenic substances.

The benefits of probiotics are not only limited to the prevention and inhibition of carcinogenic agents, but they can also include the therapeutic effect and the prevention of cancer treatment complications. The therapeutic effect of probiotics can be due to the production of antimicrobial compounds such as bacteriocins and antibiotics. Bacteriocins produced by LAB are peptides or small proteins that are frequently inhibitory towards many undesirable bacteria, including food-borne pathogens (79). It has also been suggested that LAB or a soluble compound produced by the bacteria may interact directly with tumor cells in culture and inhibit their growth (36). The competitive behavior of probiotics with pathogens is related to adhesion to epithelial cells (80). Several studies that characterized LAB from different origins has shown that the ability to adhere to epithelial cells is strain dependent (81-83). The suppressive effect of probiotics was also associated with production of short chain fatty acids (SCFAs), which could be reflected, by the enrichment of SCFAs-related pathway (84, 85). 

Chronic inflammation has been recognized as a risk factor of cancer (86). For example; inflammatory bowel disease (IBD) is a risk factor of colon cancer and the risk of HCC can be increased by inflammatory conditions, such as hepatitis B, C virus infection (87). Inflammation not only plays a role in colitis-associated colon cancer, but may also happen in sporadic colon cancer and affect the progression of cancer (88, 89). *L. rhamnosus* GG was reported to prevent colon carcinogenesis, accompanied by the suppression of NFkB pathway (90), a pro-inflammatory pathway that links IBD and colon cancer (91, 92). Li et al. (73) showed a reduction of pro-inflammatory cytokine IL-17 by probiotics in HCC model, revealing the possible relationship between immunomodulatory effect and anticancer effect of probiotics. 

## Conclusion

Probiotics have become very important in medicine because of their useful effects on the host health. Numerous in vitro studies and animal models show positive effects of probiotics on gastrointestinal cancers by various mechanisms, including anti-carcinogenic effects, anti-mutagenic properties, modification of differentiation process in tumor cells, production of short chain fatty acids, alteration of tumor gene-expressions, activation of the host’s immune system, inhibition of the bacteria that convert pro-carcinogens to carcinogens, alteration of colonic motility and transit time, as well as reduction of intestinal pH to reduce microbial activity. Different mechanisms can be involved in these beneficial effects, mainly via modulation of gut microbiota, which thereby influences host metabolism and immunity. Nevertheless, human clinical trials of the application of probiotics as bio therapeutics against cancer with adequate follow-up results are still lacking. Therefore, extensive clinical trials studies on human are required to identify the potential strains, dosages and administration regimes for specific types and stages of cancer as an alternative therapy for cancer treatment
